# Recommendations of megavoltage computed tomography settings for the implementation of adaptive radiotherapy on helical tomotherapy units*

**DOI:** 10.1002/acm2.12859

**Published:** 2020-03-26

**Authors:** Christian Velten, Robert Boyd, Kyoungkeun Jeong, Madhur K. Garg, Wolfgang A. Tomé

**Affiliations:** ^1^ Department of Radiation Oncology Montefiore Medical Center Bronx NY USA; ^2^ Albert Einstein College of Medicine Bronx NY USA

**Keywords:** adaptive radiotherapy, image quality, MVCT, tomotherapy

## Abstract

Megavoltage computed tomography (MVCT) image quality metrics were evaluated on an Accuray Radixact unit to recommend scan settings for the implementation of a consistent adaptive radiotherapy program. Megavoltage computed tomography image quality was evaluated and compared to a kilovoltage CT (kVCT) simulator using a commercial cone beam computed tomography image quality phantom. Megavoltage computed tomographies were acquired on the Accuray Radixact using fine, normal, and coarse pitches, with all available reconstruction slice thicknesses, each of which were reconstructed using standard and iterative reconstruction (IR). Image quality metrics (IQM) were evaluated using DoseLab: automatically and manually calculated spatial resolution, subject contrast, and contrast‐to‐noise ratio (CNR). Scanning time was 15.6 s/cm for fine, 8.1 s/cm for normal, and 5.6 s/cm for coarse pitch. Automatically evaluated spatial resolutions ranged from 0.39, 0.41, to 0.42 lp/mm for standard reconstruction and from 0.24, 0.21, to 0.18 lp/mm for soft‐tissue IR, respectively, with general IR yielding values in between these. Spatial resolution for kVCT was measured to be at least 0.42 lp/mm. Contrast was consistent across MVCT settings with 8.1 ± 0.2%, while kVCT contrast was 10.27 ± 0.05%. CNR was calculated to be 3.3 ± 0.4 for standard reconstruction, 7.4 ± 0.4 for general IR, and 12.0 ± 1.9 for soft‐tissue IR. It was found that increasing reconstruction slice thickness for a given pitch does not improve IQMs. Based on the consistency of contrast metrics across pitch values and the only slightly reduced spatial resolution using normal compared to fine pitch, we recommend the use of normal pitch with 2 mm slice thickness to maximize image quality for ART while limiting scanning time. Only for sites for which improved CNR is required and reduced spatial resolution is acceptable, soft‐tissue IR is recommended.

## 1 INTRODUCTION

Adaptive radiotherapy (ART) holds the promise that the on‐ or off‐line adaptation of treatment plans will improve the efficacy of cancer treatments by improving dose delivery to the tumor and minimizing normal tissue doses based on the most recent anatomy of a patient.^1^

In general, adaptation of a treatment plan due to variations in organ filling, weight gain or loss as well as tumor response requires volumetric imaging of the patient.^2^ Commonly, this is performed by acquiring a repeat simulation computed tomography (CT) scan followed by manual replanning. Automated techniques using deformable image registration (DIR) and automated segmentation of contours or contour propagation, followed by automated replanning, can make ART more feasible for use across all body sites and cancer types. These automated techniques, especially DIR and auto segmentation, require volumetric imaging with sufficient image quality to produce correct results.^3^, ^4^, ^5^, ^6^

Recently, linear accelerator platforms that include kilovoltage or megavoltage computed tomography [kilovoltage (kV)/megavoltage computed tomography (MVCT)] for daily patient setup have come into common use in clinics around the world. This volumetric imaging data could serve as the input data for automated ART. Helical tomotherapy units can perform MVCTs over the entire volume‐of‐interest (VOI) length. These MVCTs can then be used as deformation targets for the original planning kVCT, followed by structure propagation and ultimately, automated plan re‐optimization.^3^

## 2 MATERIALS AND METHODS

A commercial (cone beam) computed tomography (CT/CBCT) image quality phantom, CatPhan 504 (The Phantom Laboratory, Greenwich, NY), was used to evaluate MVCT image quality on an Accuray Radixact (Accuray, Sunnyvale, CA) radiation therapy system. This phantom contains geometry and sensitometry, high‐resolution, low‐contrast, and uniformity test modules.^7^ Other image quality phantoms with test modules for spatial resolution, contrast, and contrast‐to‐noise could also have been employed for this study.

After acquiring a kilovoltage computed tomography (kVCT) using the department's CT simulator with 2.5 mm slice thickness and 120 kVp/43 mAs technique, a phantom plan was created on the Accuray Precision treatment planning system. Megavoltage computed tomographies were then acquired on the Radixact (Accuray, Sunnyvale, CA) using fine, normal, and coarse pitches with all available reconstruction slice thicknesses: fine (1, 2 mm), normal (2, 4 mm), and coarse (3, 6 mm). Each MVCT was reconstructed using standard and iterative reconstruction (IR) techniques.^8^

Employing DoseLab v6.8 (Varian Medical Systems, Palo Alto, CA), several image quality metrics (IQM) were evaluated. Image quality metrics used in the comparison were automatically and manually evaluated spatial resolution, contrast, and contrast‐to‐noise ratio (CNR). Additionally, MVCT scanning speed in time per scan length was calculated to evaluate feasibility for daily use of MVCT imaging. Spatial resolution is given by the spatial frequency (in lp/mm) at which the value of the normalized modulation transfer function (MTF) is 50%, where the modulation values are normalized by the maximum modulation which corresponds to the lowest resolution bar pattern. Modulation is calculated as $\frac{S_{90}-S_{10}}{S_{90}+S_{10}}$ from the 90th (*S*_90_) and 10th (*S*_10_) percentile signal levels in the region‐of‐interest (ROI) defined in DoseLab. Contrast between two regions is calculated as $C_{2,1}=\frac{S_{2}-S_{1}}{S_{2}+S_{1}}$ , where *S*_2_ and *S*_1_ are the mean pixel values in the regions of larger and lower signals, respectively. CNR is calculated from the contrast between two regions, *C*_2,1_, divided by the noise present, ${\left(\frac{\sigma_{1}^{2}+\sigma_{2}^{2}}{S_{1}^{2}+S_{2}^{2}}\right)}^{1/2}$ , which is the ratio of the quadratic sums of standard deviations and signals in the ROIs.

To assess image quality outside of the axial image acquisition plane, the CBCT phantom was positioned in line with the table movement direction. Subsequently, MVCTs were acquired using all pitch values and reconstruction techniques, using the lowest available slice thickness for each. Spatial resolution and contrast metrics were evaluated from sagittal reconstructions of these images employing ImageJ following the calculation methodologies outlined above.^9^

## 3 RESULTS

**Fig. 1:**
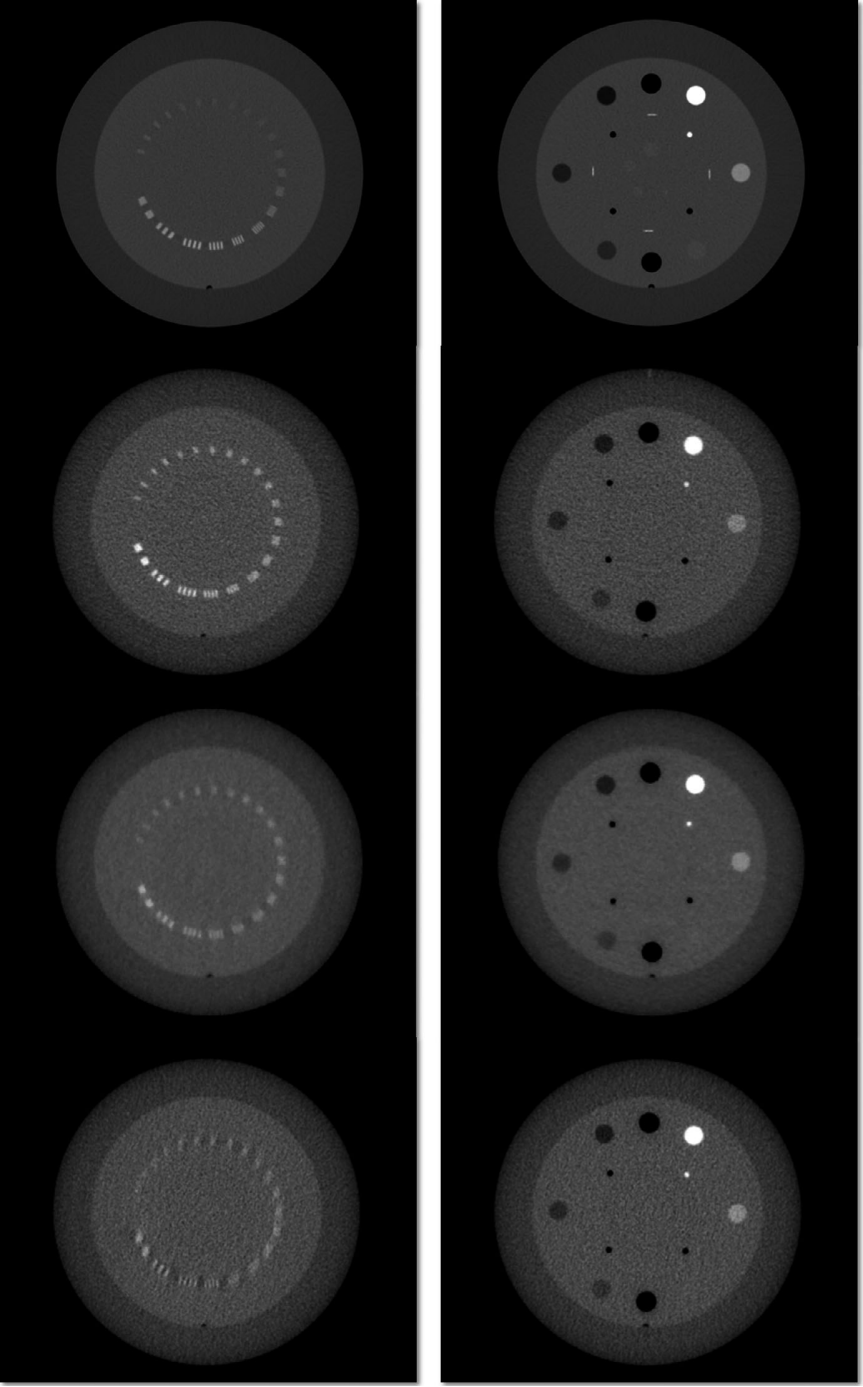
Computed tomography slices for standard protocol kilovoltage CT with 2.5 mm slice thickness (row 1) and fine, normal, and coarse pitch (rows 2–4) with standard reconstruction megavoltage CT with 1, 2, and 3 mm slice thicknesses.

**Fig. 2:**
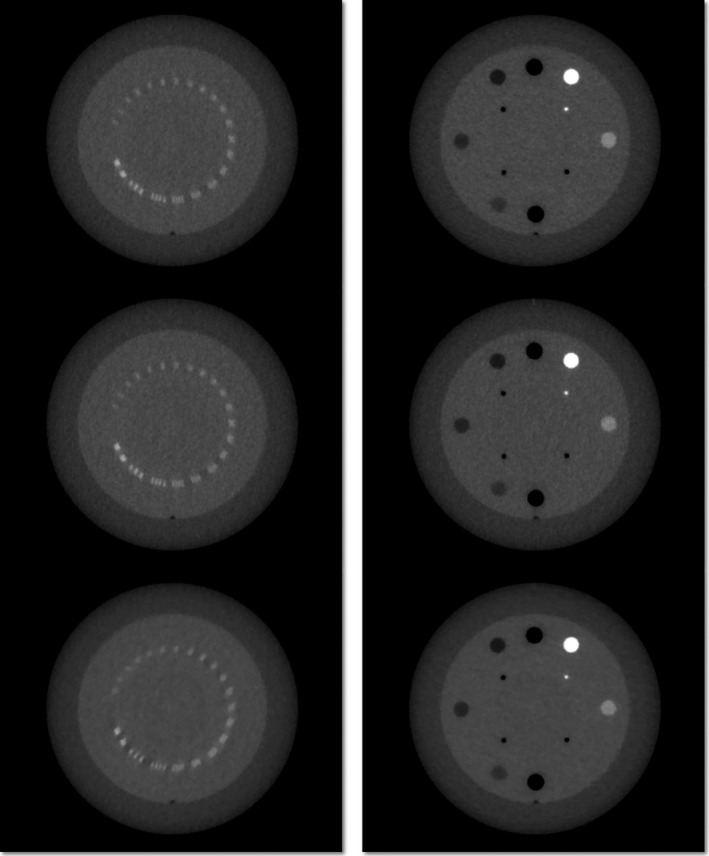
Computed tomography slices for normal pitch megavoltage computed tomography (2 mm slice thickness) using standard (row 1), general iterative (row 2), and soft‐tissue iterative (row 3) image reconstruction techniques. Spatial resolution worsens and noise decreases from standard to soft‐tissue image reconstruction, while contrast (right column) appears comparable.

Upon visual inspection, MVCT images exhibit more noise and lower spatial resolution than kVCT (cf. Fig. 1, left column, rows 1–4). While it is possible to identify the slice thickness and low‐contrast circles (inner ring) on the kVCT (cf. Fig. 1, right column, row 1), they are absent on the MVCT (cf. Fig. 1, right column, rows 2–4). The use of iterative reconstruction techniques also leads to a noticeable decrease in spatial resolution and noise at a given pitch, whereas subject contrast appears to be consistent (cf. Fig. 2).

Axial in‐plane image quality metrics for kVCT and MVCT are tabulated in Table 1. It was found that IQMs and scan rate did not substantially change with MVCT slice thickness; we thus only report the results for the smallest slice thickness at each pitch, which yield improved IQMs. Megavoltage computed tomography scanning rates depend only on the selected pitch resulting in 15.6 s/cm for fine, 8.1 s/cm for normal, and 5.6 s/cm for coarse pitch.

**Table 1:** Summary of axial in‐plane image quality metrics acquired using the kV‐cone beam computed tomography (CBCT) phantom.

Pitch	kVCT	MVCT‐fine	MVCT‐normal	MVCT‐coarse
Slice thickness (mm)	2.5	1	2	3
Scan time (s/cm)		15.6	8.1	5.6

Reconstruction		SR	IR‐G	IR‐ST	SR	IR‐G	IR‐ST	SR	IR‐G	IR‐ST
spat. res. (lp/mm)	0.45	0.39	0.28	0.24	0.41	0.29	0.21	0.42	0.19	0.18
man. Spat. res. (lp/mm)	0.70	0.50	0.45	0.40	0.50	0.40	0.30	0.40	0.20	0.20
Contrast (%)	10.2	7.9	8.1	7.8	7.8	8.1	8.2	8.6	8.0	8.1
CNR	16.1	3.3	7.0	11.4	3.0	7.8	14.2	3.7	7.3	10.5

IR‐G: general iterative image reconstruction; IR‐ST soft‐tissue image reconstruction; kVCT, kilovoltage computed tomography; MVCT, megavoltage CT; SR: Standard image reconstruction.

For fine, normal, and coarse pitches automatically detected spatial resolutions ranged from 0.39, 0.41, to 0.42 lp/mm for standard reconstruction and from 0.24, 0.21, to 0.18 lp/mm for soft‐tissue IR, respectively, with general IR yielding values in between these (cf. Fig. 2). Spatial resolution for kVCT was measured to be at least 0.42 lp/mm. In general, spatial resolution was found to be higher when evaluated manually for kVCT, as well as for a fine and normal, but not a coarse pitch MVCT. For standard reconstruction MVCTs that have been automatically evaluated, spatial resolution is comparable to kVCT (within 15%), while kVCT has superior resolution when evaluated manually (40% higher).

Contrast was found to be consistent for the different pitches and reconstruction techniques yielding an average of 8.1% ± 0.2%, which is comparable to the contrast of kVCT which was found to be 10.27% ± 0.05%. For MVCT, CNR is most consistent for similar reconstruction techniques, but varies by up to 16%, likely due to averaging effects for different slice thicknesses. We found a CNR of 3.3 ± 0.4 for standard reconstruction, 7.4 ± 0.4 for general IR, and 12.0 ± 1.9 for soft‐tissue IR. Kilovoltage computed tomography remains superior in terms of CNR, yielding a CNR of 15.6 ± 0.7. Only for soft‐tissue IR is the CNR of MVCT close to that of kVCT; however, this comes at the price of reduced spatial resolution which decreases by 50% on average (cf. Table 1).

Spatial resolution in the reconstructed sagittal plane was manually evaluated and found to be 0.3, 0.2, and 0.1 lp/mm for fine, normal, and coarse pitch, respectively. Manually evaluated subject contrast was again consistent between pitch values and standard and general iterative reconstruction techniques with an average value of 4.2% ± 0.4%; for soft‐tissue iterative reconstruction, the average contrast was 7.8% ± 0.2%. Similarly, CNR was consistent between pitch values with 2.9 ± 0.4, 3.9 ± 0.5, and 12.8 ± 2.0 for standard, general, and soft‐tissue iterative reconstruction, respectively (cf. Table 2).

**Table 2:** Summary of sagittal plane image quality metrics acquired using the kV‐CBCT phantom.

Pitch	MVCT‐fine	MVCT‐normal	MVCT‐coarse
Slice thickness (mm)	1	2	3

Reconstruction	SR	IR‐G	IR‐ST	SR	IR‐G	IR‐ST	SR	IR‐G	IR‐ST
man. Spat. res. (lp/mm)	0.30	0.30	0.30	0.20	0.20	0.20	0.10	0.10	0.10
Contrast (%)	4.6	4.2	8.0	5.1	3.5	8.0	5.4	2.68	7.43
CNR	3.5	3.5	16.0	3.0	3.3	9.2	2.2	4.8	13.3

IR‐G: general iterative image reconstruction; IR‐ST soft‐tissue image reconstruction; kVCT, kilovoltage computed tomography; MVCT, megavoltage CT; SR: Standard image reconstruction.

## 4 DISCUSSION

In this study, we have evaluated a number of image quality metrics (IQMs) for MVCT to determine which combination of slice thickness, pitch, and reconstruction algorithm will maximize the image quality at a reasonable scanning time allowing for an efficient implementation of daily imaging for ART. We found that contrast metrics were consistent across pitch values, and therefore the remaining primary IQMs for comparison were scanning speed and spatial resolution. The highest spatial and axial resolutions were found for fine pitch (using manual evaluation); however, the use of a fine pitch increases the scanning time twofold compared to the use of a normal pitch. Conversely, coarse pitch was found to reduce the spatial resolution by up to 50% for an approximately 30% saving in scanning time when compared to the scanning time for normal pitch. Our results show that a normal pitch with 2 mm slice thickness maximizes image quality while limiting scanning time and should therefore be the preferred option for implementation of ART into the clinic (cf. Table 1).

The choice of reconstruction technique depends primarily on the required soft‐tissue contrast for daily setup and DIR algorithms. In cases where bony anatomy is most adequate for registration, we recommend using standard reconstruction, which yields the highest spatial resolution with the lowest CNRs. Soft‐tissue IR is recommended if soft‐tissue contrast, that is, CNR, is tantamount and reduced spatial resolution is acceptable. General iterative reconstruction offers a middle ground between standard and soft‐tissue iterative reconstruction having both adequate spatial resolution and CNR.

Spatial resolution was found to be the most sensitive IQM for sagittal and coronal reconstructions with respect to pitch value; however, it was found to be consistent among reconstruction techniques. This is likely due to the already small spatial resolution for fine pitch in the sagittal plane at 0.3 lp/mm compared to 0.5 lp/mm in the axial plane. Furthermore, the reconstruction algorithm primarily affects IQMs in the axial slices, thus lowering their impact on IQMs in the sagittal plane which is dominated by the chosen slice thickness. Therefore, if the treated area contains important structures smaller than 10 mm, normal pitch is adequate, while structures smaller than 5 mm will necessitate the use of fine pitch.

In summary, we offer the following recommendations for the implementation of a consistent scanning protocol for adaptive dose accumulation and prospective adaptive replanning. First, daily MVCT scans should not be shorter than the largest PTV extent in the axial direction and, ideally, should include a margin superiorly and inferiorly of at least 1 cm beyond the largest PTV. Second, in order to limit treatment time slots to no more than 20 min, we suggest a maximum scanning time of 200 s, which will keep patient on couch times lower than 10–15 min in most cases. This will also help to reduce the probability for involuntary patient movement throughout imaging and treatment delivery. Third, based on our proposed scanning time restriction of 200 s, normal pitch should be used for targets having a superior–inferior extent of up to 25 cm (e.g., head‐and‐neck cancers with regional lymph node irradiation, most pelvic cancers); while a coarse pitch might be acceptable for targets that are longer than 25 cm in the superior–inferior direction. Finally, the choice of reconstruction technique should be made based on size and tissue type of important landmarks per disease site. For cranial as well as head‐and‐neck cases, spatial resolution is usually the most important IQM to ensure correct positioning given the relatively fixed location of tissue with respect to bony anatomy, thus standard reconstruction techniques are recommended. In some head‐and‐neck, as well as thoracic, mediastinal, and abdominal cases, however, where discrimination of and matching to soft tissue can be more important, the use of general iterative reconstruction or even soft‐tissue reconstruction can be warranted. Pelvic cases usually benefit from general and soft‐tissue iterative reconstruction, especially for adaptive dose accumulation and replanning, since there can be significant movement and changes in size of the involved organs‐at‐risk. In general, the reconstruction technique should be kept the same throughout the treatment to yield consistent results in adaptive dose accumulation.

## CONFLICT OF INTEREST

This work has been supported in part by a research grant from Accuray Inc. CV has received meeting attendance support from Accuray, Inc.; RB has received research support from Accuray Inc.; MG and WT have received research grants and research support from Accuray, Inc.
